# Long-Term Outcomes of Patients with Unprotected Left Main Coronary Artery Disease Treated with Percutaneous Angioplasty versus Bypass Grafting: A Meta-Analysis of Randomized Controlled Trials

**DOI:** 10.3390/jcm9072231

**Published:** 2020-07-14

**Authors:** Gani Bajraktari, Fjolla Zhubi-Bakija, Gjin Ndrepepa, Fernando Alfonso, Shpend Elezi, Zarife Rexhaj, Ibadete Bytyçi, Artan Bajraktari, Afrim Poniku, Michael Y. Henein

**Affiliations:** 1 Department of Public Health and Clinical Medicine, Umeå University, 90737 Umeå, Sweden; i.bytyci@hotmail.com (I.B.); artanbajraktari@hotmail.com (A.B.); michael.henien@umu.se (M.Y.H.); 2 Clinic of Cardiology, University Clinical Centre of Kosova, 10000 Prishtina, Kosovo; fjolla.zhubi@gmail.com (F.Z.-B.); dr.zariferexhaj@gmail.com (Z.R.); afrimponiku@hotmail.com (A.P.); 3 Medical Faculty, University of Prishtina “Hasan Prishtina”, 10000 Prishtina, Kosovo; selezi@hotmail.com; 4 Deutsches Herzzentrum München, Technische Universität, 80636 Munich, Germany; ndrepepa@dhm.mhn.de; 5 Cardiac Department, La Princesa University Hospital, Institute of Health Research, IIS-IP, CIBER-CV University Autónoma of Madrid, 28029 Madrid, Spain; falf@hotmail.com

**Keywords:** coronary artery disease, unprotected left main, percutaneous coronary intervention, coronary artery bypass graft

## Abstract

Background and Aim: Treatment of patients with left main coronary artery disease (LMCA) with percutaneous coronary intervention (PCI) or coronary artery bypass grafting (CABG) remains controversial. The aim of this meta-analysis was to compare the long-term clinical outcomes of patients with unprotected LMCA treated randomly by PCI or CABG. Methods: PubMed, MEDLINE, Embase, Scopus, Google Scholar, CENTRAL and ClinicalTrials.gov database searches identified five randomized trials (RCTs) including 4499 patients with unprotected LMCA comparing PCI (*n* = 2249) vs. CABG (*n* = 2250), with a minimum clinical follow-up of five years. Random effect risk ratios were used for efficacy and safety outcomes. The study was registered in PROSPERO. The primary outcome was major adverse cardiac events (MACE), defined as a composite of death from any cause, myocardial infarction or stroke. Results: Compared to CABG, patients assigned to PCI had a similar rate of MACE (risk ratio (RR): 1.13; 95% CI: 0.94 to 1.36; *p* = 0.19), myocardial infarction (RR: 1.48; 95% CI: 0.97 to 2.25; *p* = 0.07) and stroke (RR: 0.87; 95% CI: 0.62 to 1.23; *p* = 0.42). Additionally, all-cause mortality (RR: 1.07; 95% CI: 0.89 to 1.28; *p* = 0.48) and cardiovascular (CV) mortality (RR: 1.13; 95% CI: 0.89 to 1.43; *p* = 0.31) were not different. However, the risk of any repeat revascularization (RR: 1.70; 95% CI: 1.34 to 2.15; *p* < 0.00001) was higher in patients assigned to PCI. Conclusions: The findings of this meta-analysis suggest that the long-term survival and MACE of patients who underwent PCI for unprotected LMCA stenosis were comparable to those receiving CABG, despite a higher rate of repeat revascularization.

## 1. Introduction

The available evidence supporting the treatment of patients with left main coronary artery disease (LMCA) in support of percutaneous coronary intervention (PCI) or coronary artery bypass grafting (CABG) remains unascertained. Current clinical guidelines recommend PCI as an appropriate alternative to the standard treatment with CABG in patients with LMCA and low-to-intermediate anatomical complexity [1,2]. Randomized clinical trials (RCTs) with long follow-up results [3,4,5,6] have recently been published showing comparable results for the two procedures, with a more frequent need for repeat revascularization in patients treated with PCI. Additional data are required to overcome the limitation of the sample size in individual RCTs in comparing the primary clinical outcome endpoints, including death, stroke, myocardial infarction and the need for revascularization, between the two treatment strategies. Since atherosclerotic disease is progressive in nature, an assessment of the outcomes of coronary interventions at long-term follow-ups should be highly desirable. To further strengthen the evidence, we sought to meta-analyze all available RCTs that compared the clinical outcome of PCI and CABG treatments of unprotected LMCA reporting long-term (≥five years) clinical follow -up data on the two treatment strategies. If the same results hold, they may then have a significant impact on future clinical guidelines updates.

## 2. Methods

We followed the guidelines of the 2009 Preferred Reporting Items for Systematic Reviews and Meta-Analysis (PRISMA) statement [7]. Due to the study design (meta-analysis), neither the Institutional Review Board (IRB) approval nor patient informed consent were needed.

### 2.1. Search Strategy

We systematically searched PubMed-Medline, EMBASE, Scopus, Google Scholar, the Cochrane Central Registry of Controlled Trials and ClinicalTrial.gov up to May 2020 using the following key words: (“left main stem” OR “left main coronary artery disease”) AND (“percutaneous coronary intervention” OR “PCI”) AND (“coronary artery bypass grafting” OR “CABG”). Other potentially suitable trials for inclusion in the analysis included abstracts from selected congresses: Scientific Sessions of the American Heart Association (AHA), European Society of Cardiology (ESC), American College of Cardiology (ACC) and European Society of Atherosclerosis (EAS). Only articles published in English were included. No filters were applied. G.B. and F.Z.B. independently evaluated all articles separately. The finally selected articles were obtained in full text and searched carefully by the same two researchers independently. They extracted the necessary data and evaluated the articles’ quality. Disagreements were resolved by discussion with a third party (M.Y.H.).

### 2.2. Eligibility Criteria

Studies eligible for inclusion were those fulfilling the following criteria: (1) randomized design comparing the efficacy and safety of PCI with that of CABG in patients with unprotected LMCA disease, (2) minimum follow-up of 5 years and (3) full-text studies published in peer-reviewed journals. Exclusion criteria were: (1) nonrandomized studies, (2) follow-up less than 5 years, (3) unpublished papers and (4) ongoing trials. Observational and unpublished studies were not included in the meta-analysis.

### 2.3. Data Extraction

Qualified studies were searched, and the following data were collected, including: (a) first author’s name, (b) date of publication, (c) clinical trial name, (d) place where the study was conducted, (e) number of centers involved, (f) study design, (g) number of patients in each of the two study arms who received LMCA treatment, (h) follow-up period and (i) detailed clinical outcome and nature of events in the two groups.

### 2.4. Outcomes and Definitions

The primary outcome of the analysis was the major adverse cardiac events (MACE), defined as the composite of death from any cause, stroke or myocardial infarction. The secondary outcomes tested in this meta-analysis were all-cause mortality, cardiovascular (CV) mortality, nonfatal myocardial infarction and any revascularization or stroke.

### 2.5. Quality Assessment

Risk of bias assessment in the included studies was evaluated by the same investigators for each study and was performed systematically using the Cochrane quality assessment tool for RCTs [8]. Tests for publication bias were not used, as it is recommended to be performed in the event of 10 or more trials being included for analysis [9]. We used the known seven criteria for quality assessment according to the Cochrane tool, including: allocation sequence concealment (selection bias), random sequence generation (selection bias), blinding of outcome assessment (detection bias), blinding of participants and personnel (performance bias), incomplete outcome data (attrition bias), selective outcome reporting (reporting bias) and other potential sources of bias. The risk of bias in each study was conventionally classified as being “low”, “high” or “unclear” (Supplementary Table S1).

### 2.6. Statistical Analysis

We performed the pooled analyses of treatment effects and clinical outcomes using the Cochrane Collaborative software, RevMan 5.3.5 (the Nordic Cochrane Center, the Cochrane Collaboration, 2014, Copenhagen, Denmark) [10]. Baseline characteristics were reported as median and range. Mean and standard deviation (SD) values were estimated using the method described by Hozo et al. [11]. Analysis was presented in forest plots. A two-tailed *p*-value < 0.05 was considered significant. Meta-analyses were performed using the fixed-effects model, and the random effect model was used if heterogeneity was encountered. Heterogeneity between studies was assessed using the Cochrane Q test and I^2^ index, with I^2^ < 25% indicating low, 25–50% moderate and >50% high heterogeneity [12]. Based on a hazard ratio value of 1, above or below, we calculated the relative risk for cardiovascular events [13]. Publication bias was assessed using Egger’s test and the visual inspection of funnel plots.

## 3. Results

### 3.1. Search Results and Trial Flow

**Figure 1:**
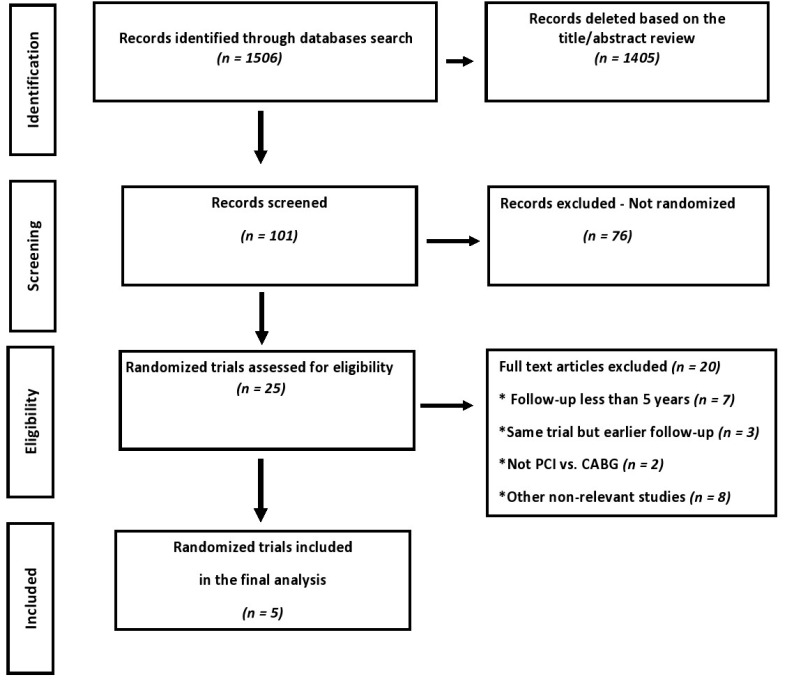
Preferred Reporting Items for Systematic Reviews and Meta-Analysis (PRISMA) study selection flow chart. PCI: percutaneous coronary intervention and CABG: coronary artery bypass grafting.

Of 1506 articles identified in the initial search, 101 studies were screened as potentially relevant, but following critical scrutiny, only five RCTs [3,4,5,6,14,15] were considered appropriate and were included in this meta-analysis (Figure 1).

### 3.2. Characteristics of Included Studies

Of the 4499 patients eligible for analysis, 2249 patients were assigned to PCI and 2250 assigned to CABG. From the five included RCTs, three reported 10-year follow-ups, and the remaining two RCTs reported data on five-year follow-ups. The mean follow-up of the five RCTs was eight years (range 5–10 years). Random-effect risk ratios were used for efficacy and safety outcomes. The mean age of the patients was 65 years. The main characteristics of the included studies are reported in Table 1.

### 3.3. Primary Clinical Outcomes

#### MACE

**Figure 2:**
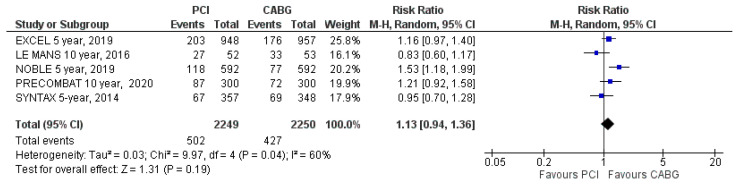
Risk of major adverse cardiac events (MACE) at the latest follow-up: PCI vs. CABG.

MACE was reported in all five included trials. MACE occurred in 502 patients (22%) assigned to PCI and in 427 patients (19%) assigned to CABG (RR: 1.13; 95% CI: 0.94 to 1.36; *p* = 0.19; Figure 2) at the latest follow-up. MACE was not statistically different between PCI and CABG treatment groups. There was high heterogeneity (I^2^ = 60%).

### 3.4. Secondary Clinical Outcomes

#### 3.4.1. All-Cause Mortality

**Figure 3:**
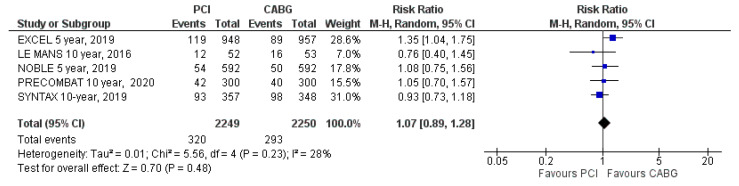
Risk of all-cause mortality at the latest follow-up: PCI vs. CABG.

All-cause mortality was reported in all trials. Mortality from any cause occurred in 320 patients (14%) assigned to PCI and in 293 patients (13%) assigned to CABG (RR = 1.07; 95% CI: 0.89 to 1.28; *p* = 0.48, Figure 3) at the latest follow-up. There was no difference in the occurrence of all-cause mortality between the two treatment strategies. There was moderate heterogeneity (I^2^ = 28%).

#### 3.4.2. CV Mortality

**Figure 4:**
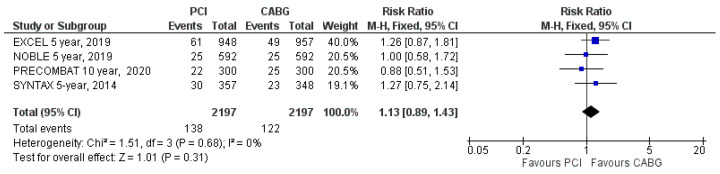
Risk of cardiovascular mortality at latest follow-up: PCI vs. CABG.

CV mortality was reported in four out of five RCTs. CV mortality occurred in 138 patients (6.3%) assigned to PCI and in 122 patients (5.6%) assigned to CABG (RR: 1.13; 95% CI: 0.89 to 1.43; *p* = 0.31; Figure 4) at the latest follow-up. The difference in CV mortality was not significant between the two groups. There was no heterogeneity (I^2^ = 0%).

#### 3.4.3. Stroke

**Figure 5:**
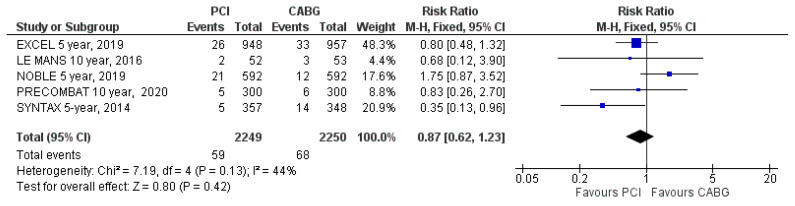
Risk of stroke at the latest follow-up: PCI vs. CABG.

Stroke was reported in all five included trials. Stroke occurred in 59 patients (2.6%) assigned to PCI and in 68 patients (3%) assigned to CABG (RR: 0.87; 95% CI: 0.62 to 1.23; *p* = 0.42; Figure 5) at the latest follow-up. The incidence of stroke during follow-up did not differ significantly between the two treatment groups. There was moderate heterogeneity (I^2^ = 44%).

#### 3.4.4. Myocardial Infarction

**Figure 6:**
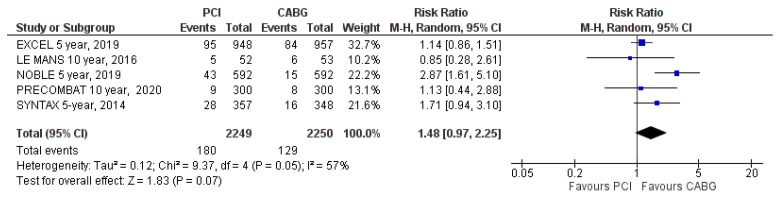
Risk of myocardial infarction at the latest follow-up: PCI vs. CABG.

Myocardial infarction was reported in all five included trials. Myocardial infarction occurred in 180 patients (8%) assigned to PCI and in 129 patients (5.7%) assigned to CABG (RR: 1.48; 95% CI: 0.97 to 2.25; *p* = 0.07; Figure 6) at the latest follow-up. The difference was not significant, and there was high heterogeneity (I^2^ = 57%).

#### 3.4.5. Unplanned Repeat Revascularization

**Figure 7:**
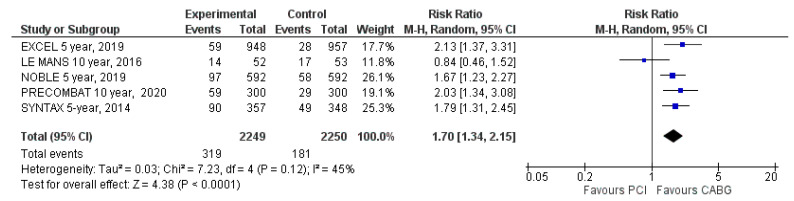
Risk of repeat revascularization at the latest follow-up: PCI vs. CABG.

Data on unplanned repeat revascularizations were reported in all five included trials. Unplanned repeat revascularizations occurred in 319 patients (14%) assigned to PCI and in 181 patients (8%) assigned to CABG (RR: 1.70; 95% CI: 1.34 to 2.15; *p* < 0.001; Figure 7) at the latest follow-up. Repeat revascularizations were significantly more frequent among patients treated with PCI than CABG. There was moderate heterogeneity (I^2^ = 45%).

There was no evidence for publication bias according to the Egger’s test used for any of the outcomes assessed.

#### 3.4.6. Risk of Bias Assessment

The quality of all included studies was assessed for a risk of bias and applicability concerns by applying the Quality Assessment of Diagnostic Accuracy Studies questionnaire (QUADAS-2) principles (Supplementary Table S2) [7]. All criteria domains for the risk of bias and applicability were analyzed. The risk of bias was classified as “low”, “high” or “unclear”. Most studies had high quality and clearly defined objectives and main outcomes (Supplementary Figure S1). All domains had a low risk of bias (<20%) and no evidence for publication bias basis.

## 4. Discussion

### 4.1. Findings

This meta-analysis of RCTs compared the long-term efficacy of a PCI strategy compared with a CABG strategy in treating symptomatic patients with unprotected LMCA. The main findings at a median follow-up of eight years can be summarized as follows: (1) There was no significant difference in the occurrence of MACE, all-cause mortality, cardiac death, myocardial infarction and stroke between patients assigned to PCI compared with those assigned to a CABG treatment strategy. (2) PCI was associated with higher rates for a need for unplanned revascularization during follow-up compared with CABG.

### 4.2. Data Interpretation

This is the first meta-analysis that included only RCTs with a long-term follow-up with a minimum of five years of patients receiving revascularization for unprotected LMCA disease. The traditional well-established treatment of LMCA has been CABG with left internal mammary artery grafted to the left anterior descending coronary artery. This approach, over the years, has proved to provide excellent clinical outcomes to these challenging patients [16]. With the recent development of excellent quality drug-eluting stents [17], interest developed to implant them in patients with high-grade LMCA stenosis—particularly those carrying significant surgical risk. In keeping with previous publications [18,19] in the same field, our results showed that the long-term efficacy of PCI for treating unprotected LMCA disease is similar to CABG, with similar rates of MACE and secondary clinical outcomes, even all-cause and cardiovascular mortality. However, such similarities of hard endpoints were on the expense of PCI patients requiring more often repeat revascularizations than the surgical ones. This difference was not surprising for a number of reasons. Firstly, the stented coronary segment remains a vulnerable spot for atherosclerotic disease progression within and/or outside the stented segment [20]. In contrast, with CABG coronary flow is guaranteed through the LIMA, known to be strikingly resistant to the development of obstructive atherosclerosis [21]. Secondly, atherosclerosis is a progressive disease [22]; therefore, despite repairing the LMCA stenosis, the underlying pathology may progress in some patients over the course of the follow-up period, even despite efforts to control the risk factors. With CABG, unless the progressive disease is distal to the graft site or in another major branch, patients are expected to maintain good coronary circulation. Thirdly, the same principle applies to those with extensive coronary calcification, in whom calcium begets calcium [23]. A localized area of recurrent severe LMCA calcification in PCI patients would require repeat revascularizations compared to a LIMA that is distally grafted, thus bypassing all proximal blood flow obstacle lesions.

The results of this study have significant importance when compared to other recently published studies. Distinct from previous meta-analyses, we included only RCTs with minimum follow-up periods of five years, and consequently, the outcomes for both strategies were assessed over a median of eight-year follow-ups. This expanded the follow-up period compared with the recently published meta-analyses of RCTs that reported the outcomes in patients treated with PCI or CABG over five to six years of clinical follow-up [18,19]. The longer follow-up period provided by our meta-analysis allowed a better assessment of the impact of the disease progression in these patients. However, even with a longer follow-up, PCI showed a comparable efficacy with that of CABG for most clinical outcomes [24].

### 4.3. Clinical Implications

This meta-analysis clearly demonstrated comparable long-term clinical outcomes for PCI compared with CABG treatments of patients with unprotected LMCA disease. Hard clinical outcomes, including mortality, were not different between the two treatment strategies, but the higher need for repeat revascularizations with the nonsurgical procedure should be considered in the decision-making process. It is expected that, with the continuous development of better-quality stents, resulting in better arterial stability and tissue compatibility and a lower need for repeat revascularizations, PCI might become the established treatment for select patients with LMCA disease.

### 4.4. Limitations

The available number of RCT eligible for inclusion in this meta-analysis was small, particularly because of the required minimum five-year follow-up. However, despite the extended follow-up period, the main results are comparable to previously published meta-analyses [18,19]. As is the case with all study-level meta-analyses, we did not have control of the patients’ recruitment strategies in the two arms of each RCT; individual investigators of these RCTs followed strict protocols. Some of the secondary clinical outcomes were not available in the five RCTs, but this did not impact the overall results, as these were consistent with previously published results in studies with shorter clinical follow-ups.

## 5. Conclusions

The findings of this meta-analysis, with the longest clinical follow-up currently available, suggest that the MACE rate and long-term survival of patients were comparable in patients receiving PCI or CABG for unprotected left main stem disease. However, the rate of repeat revascularizations was higher in patients treated with PCI.

## Figures and Tables

**Table 1:** Trials characteristics.

	Excel	Le Mans	Noble	Precombat	Syntax
**Publication year**	2019	2016	2019	2020	2019
**Number of patients after 5-year follow-ups**	1905 (PCI *n* = 948; CABG *n =* 957)	105 (PCI *n =* 52; CABG *n =* 53)	1184 (PCI *n =* 592; CABG *n =* 592)	600 (PCI *n =* 300; CABG *n =* 300)	705 (PCI *n =* 357; CABG *n =* 348)
**Major inclusion criteria**	Unprotected LMCA stenosis >70% or >50% if hemodynamically significant	Symptomatic stenosis > 50% of unprotected LMCA	Unprotected LMCA stenosis > 50% or FFR ≤ 0.80 without more than three noncomplex lesions	Symptomatic or asymptomatic unprotected LMCA stenosis > 50% regardless of other significant lesions	Symptomatic stenosis > 50% of unprotected LMCA or with assessed myocardial ischemia
**Major exclusion criteria**	PCI or CABG of unprotected LMCA in the previous year, need of concomitant cardiac surgery, SYNTAX score ≥33 or life expectancy < 3 years	Previous MI, total occlusion of the left main, Euroscore surgical risk of 8 or more, stroke or transient ischemic attack within 3 months, renal dysfunction or contraindication to antiplatelet therapy	Patients considered too high-risk for PCI or CABG, STEMI < 24 h or life expectancy < 1 year	MI in the previous week, PCI in the previous year, LVEF < 30%, cardiogenic shock, stroke in the previous 6 months, CKD, severe hepatic dysfunction or life expectancy < 1 year	Previous PCI or CABG, acute MI or need for concomitant cardiac surgery

CABG: coronary artery bypass graft, CKD: chronic kidney disease, FFR: fractional flow reserve, LVEF: left ventricular ejection fraction, MI: myocardial infarction, PCI: percutaneous coronary intervention, STEMI: ST-elevation myocardial infarction and TVR: target vessel revascularization. NOBLE = The Nordic–Baltic–British Left Main Revascularization trial, SYNTAX = The Synergy between PCI with Taxus and Cardiac Surgery trial, EXCEL = Evaluation of XIENCE versus Coronary Artery Bypass Surgery for Effectiveness of Left Main Revascularization trial, PRECOMBAT = Premier of Randomized Comparison of Bypass Surgery versus Angioplasty Using Sirolimus-Eluting Stent in Patients with Left Main Coronary Artery Disease trial and LE MANS = Left Main Coronary Artery Stenting trial.

## Supplementary Materials

The following are available online at https://www.mdpi.com/2077-0383/9/7/2231/s1, Supplementary Table S1: Qualitative assessment of study reporting, Supplementary Table S2: Summary of QUADAS-2 Assessment of Selected Studies, Supplementary Figure S1: Risk of bias.
