# Supplementary material for: Long-Term Outcomes of Patients with Unprotected Left Main Coronary Artery Disease Treated with Percutaneous Angioplasty versus Bypass Grafting: A Meta-Analysis of Randomized Controlled Trials
Source: J Clin Med. 2020 Jul 14;9(7):2231. doi: 10.3390/jcm9072231 (PMC7408984; doi:10.3390/jcm9072231)

Supplementary Table 1. Qualitative assessment of study reporting

Domain	Questions	Judgments
**Risk of bias**		
1) Patient selection	*Was a consecutive or random sample of patients enrolled?*	Yes, No, Unclear
	Was a case-control design avoided?	Yes, No, Unclear
	Did the study avoid inappropriate exclusions?	Yes, No, Unclear
	Could the selection of patients have introduced bias?	Low, High, Unclear
**Applicability** 1) Patient selection	*Is there concern that the included patients do not match the review questions?*	Low, High, Unclear
**Risk of bias** 2) Index test	*Were the index test results interpreted without knowledge of the results of the reference standard?*	Yes, No, Unclear
	If a threshold was used, was it pre-specified?	Yes, No, Unclear
	Could the conduct or interpretation of the index test have introduced bias?	Low, High, Unclear
**Applicability** 2) Index test	*Is there concern that the index test, its conduct, or interpretation differ from the review question?*	Low, High, Unclear
**Risk of bias** 3) Reference standard	*Is the reference standard likely to correctly classify the target condition?*	Yes, No, Unclear
	Were the reference standard results interpreted?	Yes, No, Unclear
	without knowledge of the results of the index test?	Yes, No, Unclear
	Could the reference standard, its conduct, or its interpretation have introduced bias?	Low, High, Unclear
**Applicability** 3) Reference standard	*Is there concern that the target condition as defined by the reference standard does not match the review question?*	Low, High, Unclear
**Risk of bias** 4) Flow and timing	*Was there an appropriate interval between index test(s) and reference standard?*	Yes, No, Unclear

	Did all patients receive a reference standard?	Yes, No, Unclear
	Did patients receive the same reference standard?	Yes, No, Unclear
	Were all patients included in the analysis?	Yes, No, Unclear
	Could the patient flow have introduced bias?	Low, High, Unclear

Supplementary Table 2. Summary of QUADAS-2 Assessment of Selected Studies

Author (year) reference	Risk of bias				Applicability concerns		
	Patients selection	Index test	Reference standard	Flow and timing	Patients selection	Index test	Reference standard
**EXCEL (2019)**	Low	Low	Low	Low	Low	Low	Low
**LE MANS (2016)**	Low	Low	Low	Unclear	Low	Low	Low
**NOBLE (2019)**	Low	Low	Low	Low	Low	Low	Low
**PRECOMBAT (2020)**	Low	Low	Low	Unclear	Low	Low	Low
**SYNTAX (2019)**	Low	Low	Low	Low	Low	Low	Low

QUADAS-2: Quality Assessment of Diagnostic Accuracy Studies-2.

Supplementary Figure 1. Risk of bias

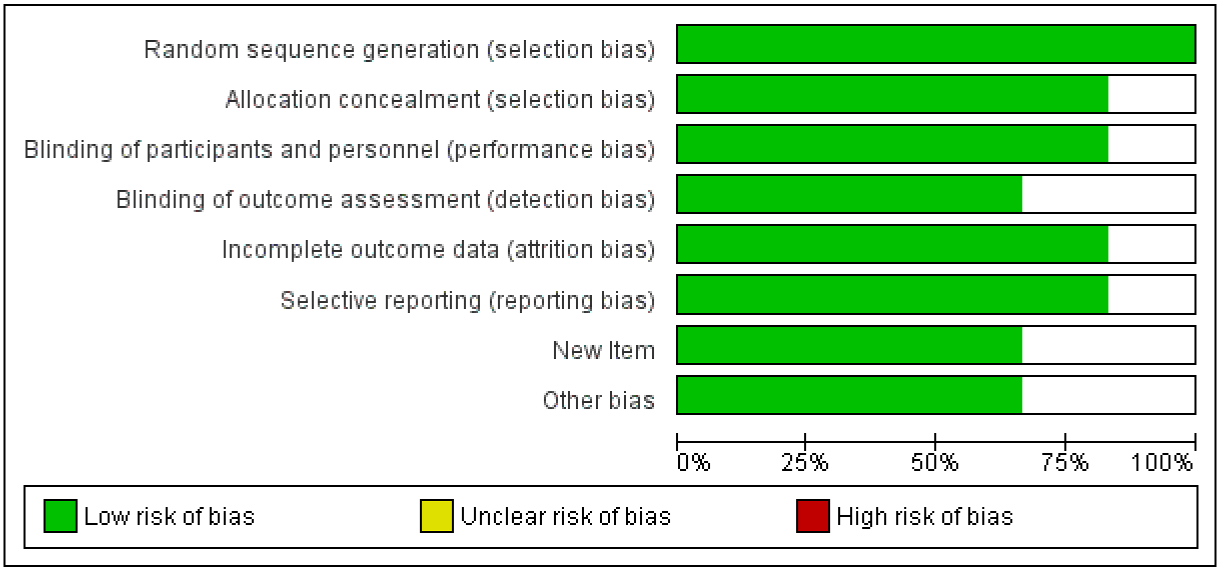

Supplement: Supplementary file 1 [file jcm-09-02231-s001.pdf]
